# IL-6 and ITS correlation to stress hyperglycemia in diabetic and non-diabetic critically ILL septic patients

**DOI:** 10.1186/2197-425X-3-S1-A304

**Published:** 2015-10-01

**Authors:** AM Fayed, MM El-sawy, AA-E Mahrous, ME Soliman

**Affiliations:** Alexandria University, Faculty of Medicine, Critical Care Medicine, Alexandria, Egypt; Alexandria University, Faculty of Medicine, Clinical Pathology, Alexandria, Egypt

## Introduction

Hyperglycemia has long been recognized as a common occurrence in critically ill patients, even without a history of diabetes resulting from the acute metabolic and hormonal changes associated with the response to injury and stress

## Objectives

To assess the correlation between blood level of IL-6 and the severity of hyperglycemia on ICU admission,glucose control, duration on mechanical ventilation, length of ICU and hospital stay in both diabetic and non-diabetic septic patients.

## Methods

The study was carried out on 87 (59 diabetic and 28 non-diabetic)adult patients who were admitted to the critical care units in Alexandria Main University Hospital with severe sepsis and septic shock stayed in the ICU for 72 hour or longer,did not receive steroid therapy prior to ICU admission, and does not had a history of malignancy.

## Results

A positive correlation between high levels of IL-6 on and blood glucose level on ICU admission in all studied cases, which was much stronger in non-diabetic group (n = 28, r = 0.986, p < 0.001) than diabetic group (n = 59, r = 0.629, p = 0.001), · the insulin dose, the duration needed to reach target glucose level (140-180 mg/dl) and the mean IL-6 level on admission in all studied patients, this positive correlation was much stronger in non- diabetic group (p < 0.001) than in diabetic group (p = 0.002,0.003) respectively, also the duration on mechanical ventilation, the length of ICU and hospital stay was positively correlated to high IL-6 levels on admission.

## Conclusions

High IL-6 level is correlated with hyperglycemia on admission and difficulties in glucose control. These results suggest there is a possibility that hypercytokinemia might be involved in the development of hyperglycemia in sepsis, and that it might thereby affect the success of glucose control, IL-6 level is correlated to the length of both ICU and hospital stay in critically ill septic patients, these results confirm the prognostic value of IL-6 in septic patients.

